# Targeted degradation of MERTK and other TAM receptor paralogs by heterobifunctional targeted protein degraders

**DOI:** 10.3389/fimmu.2023.1135373

**Published:** 2023-07-20

**Authors:** Varsha Gadiyar, Gopi Patel, Jesse Chen, Dominico Vigil, Nan Ji, Veronica Campbell, Kirti Sharma, Yatao Shi, Matthew M. Weiss, Raymond B. Birge, Viralkumar Davra

**Affiliations:** ^1^ Department of Microbiology, Biochemistry and Molecular Genetics, Cancer Center, Rutgers- New Jersey Medical School, Newark, NJ, United States; ^2^ Department of Research and Development, Kymera Therapeutics, Watertown, MA, United States

**Keywords:** TAM receptors, MERTK, AXL, TYRO3, receptor down-regulation, heterobifunctional targeted protein degraders

## Abstract

TAM receptors (TYRO3, AXL, and MERTK) comprise a family of homologous receptor tyrosine kinases (RTK) that are expressed across a range of liquid and solid tumors where they contribute to both oncogenic signaling to promote tumor proliferation and survival, as well as expressed on myeloid and immune cells where they function to suppress host anti-tumor immunity. In recent years, several strategies have been employed to inhibit TAM kinases, most notably small molecule tyrosine kinase inhibitors and inhibitory neutralizing monoclonal antibodies (mAbs) that block receptor dimerization. Targeted protein degraders (TPD) use the ubiquitin proteasome pathway to redirect E3 ubiquitin ligase activity and target specific proteins for degradation. Here we employ first-in-class TPDs specific for MERTK/TAMs that consist of a cereblon E3 ligase binder linked to a tyrosine kinase inhibitor targeting MERTK and/or AXL and TYRO3. A series of MERTK TPDs were designed and investigated for their capacity to selectively degrade MERTK chimeric receptors, reduce surface expression on primary efferocytic bone marrow-derived macrophages, and impact on functional reduction in efferocytosis (clearance of apoptotic cells). We demonstrate proof-of-concept and establish that TPDs can be tailored to either selectivity degrades MERTK or concurrently degrade multiple TAMs and modulate receptor expression *in vitro* and *in vivo*. This work demonstrates the utility of proteome editing, enabled by tool degraders developed here towards dissecting the therapeutically relevant pathway biology in preclinical models, and the ability for TPDs to degrade transmembrane proteins. These data also provide proof of concept that TPDs may serve as a viable therapeutic strategy for targeting MERTK and other TAMs and that this technology could be expanded to other therapeutically relevant transmembrane proteins.

## Graphical Abstract

Schematic representation of mechanism of heterobifunctional TAM degraders. Novel degraders consist of TAM binding moiety linked to a novel ligand that engages cereblon. This bivalent molecule allows for the ubiquitylation and proteasomal degradation of the TAMs by the UPS (Ubiquitin Proteasome System).
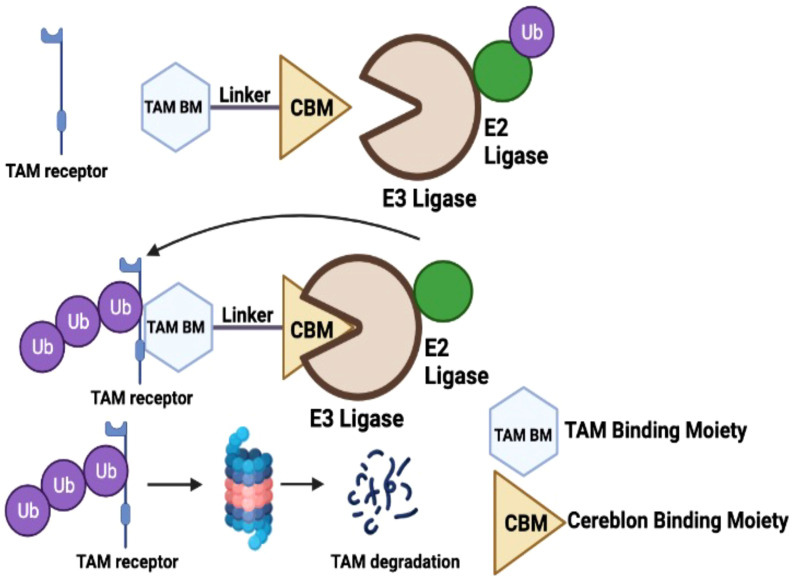

## Introduction

Receptor tyrosine kinases (RTKs) are type I cell surface receptors that regulate a number of critical physiological processes under homeostatic conditions (1, 2). However, as a result of gene amplification, overexpression, and activating mutations, RTKs are often associated with cancer initiation, progression, and metastasis, and are important targets as cancer therapeutics (3–5) TAM receptors (TYRO3, AXL, and MERTK) are a homologous family of type I RTKs that share homologous extracellular domains comprising two immunoglobulin-like domains (Ig) and two fibronectin type III domains (FN-type III) as well as a conserved KW(I/L)A(I/L)ES sequence in the kinase domain that is unique to TAM RTKs (6–8), the latter providing potential specificity for small molecule kinase inhibitors. Although overexpression of TAM receptors are widely observed in multiple malignancies and implicated in cancer progression and advanced disease, knockout of TAMs does not result in embryonic lethality, suggesting they are not essential kinases (9–11) As such, targeting TAMs in cancer has gained traction in recent years as they are predicted to have reduced off-target toxicities and hence manifest efficacy with more favorable therapeutic index (12–14).

In addition to their role as oncogenic tyrosine kinases, all three TAMs have concomitantly emerged as potential targets with immuno-oncology applications, as TYRO3, AXL, and MERTK are all expressed on immune cells, including macrophages, dendritic cells (DCs), myeloid derived suppressor cells (MDSCs), natural killer (NK) and natural killer- T (NKT) cells, where they are generally associated with inhibitory functions that suppress host anti-tumor immunity (7, 15–18). In recent years, a panoply of tyrosine kinase inhibitors has been developed, with goals to either selectively target TAMs in cancer cells, including drug resistant cancer cells, as well as simultaneously target TAMs in the immune microenvironment, the latter often with observed synergy with immune checkpoint inhibitors (19–24). Bemcentinib (BGB324/R428), for example, a selective AXL inhibitor widely used in several advanced or metastatic applications, has been observed to decrease tumor cell migration and invasion *in vitro*, and impair tumor growth and metastasis *in vivo* (25–27). More recently, it’s use has been expanded for both preclinical and clinical utility in combination with Pembrolizumab in advanced non-small cell lung cancer (28) as well as triple negative breast cancer and inflammatory breast cancer (clinical trials.gov NCT03184558).

Other small molecule inhibitors, such as ONO-7475 and INCB081776 (AXL and MERTK) (19, 20), have demonstrated promising results in advanced and metastatic solid tumors as “pan-TAM” kinase inhibitors, and also act in combination with EGFR inhibitors in EGFR-mutated NSCLC (ONO-7475 29), or synergistically with anti-PD1 to enhance immune responses and increased intra-tumoral CD4+/CD8+ T cells (INCB081776) (30, 31). Examples of TAM inhibitors that target one or more TAMs with broader spectrum tyrosine kinase specificity include MRX-2843 (MERTK and FLT3) (32, 33), BPI-9016M (AXL and MET) (34) Q702 (AXL, MERTK, CSF1R) (35), RXDX-106 (c-MET/pan-TAM) (36), and BMS 777607 (pan-TAM/FLT-3, RON) (37) and demonstrate utility in advanced and metastatic leukemic and solid tumors. Aforementioned approaches with an apparent multi-target strategy have the potential to inhibit combinations of oncogenic kinases to enhance therapeutic efficacies. Consistent with this idea, MRX-2843 retains activity in Quizartinib-resistant FLT-ITD mutant proteins with clinically relevant AML mutations, suggesting a more complex mechanism of dual MERTK/FLT3 oncogene inhibition (33). RXDX-106, an oral small molecule c-MET/pan-TAM inhibitor (36) has utility in both breast cancer and colorectal models not only to impair tumor growth *in vivo*, but also increased tumor infiltrating leukocytes and IFN-γ production, as well as M1 intra-tumoral macrophages and NK cells at the tumor microenvironment (36). BMS-777607, a tyrosine kinase inhibitor that was originally designed as a MET inhibitor but also broadly targets TYRO3, AXL, and MERTK as a pan-TAM inhibitor, serves to target AXL expressed on tumor cells while also targeting MERTK expressed on macrophages, the latter inhibiting macrophage efferocytosis and imparting tumor immunity that can synergize with anti-PD1 mAb (37). Collectively, these studies provide important direct and anecdotal evidence suggesting that TAM tyrosine kinase inhibitors have complex and pleotropic actions in tumor biology, with efficacy in a number of tumor-specific applications depending on tumor type, mutation burden and drug resistance, as well as the local immune microenvironment.

Here we report on a proof-of-concept investigational strategy to exploit a TPD approach to modulate levels of TAMs. The heterobifunctional molecules utilized in this work consist of a novel ligand for the E3 ligase cereblon and MERTK ligands inspired by work carried out by researchers at the University of North Carolina (UNC). The conjugation of these ligands with a linker result in heterobifunctional molecules capable of directing the ubiquitin-mediated proteolysis of MERTK and other TAMs. Unlike small molecule inhibitors that depend on sustained target engagement to elicit the desired pharmacology, heterobifunctional degraders initiate an event driven process, and this removal of the targeted protein can in turn lead to sustained pharmacology. TPDs are also expected to reduce compensatory upregulation of receptor expression due to the chaperone mediated stabilization of kinases (38, 39) as well as compensatory upregulated kinase inactive receptors (40). For example, studies by Lauter et al. demonstrated that kinase inhibited AXL feedbacks to increase steady-state AXL expression (40). Leveraging this targeted protein degradation approach, we provide evidence for both selective degradation of MERTK (KTX-335), as well as broader MERTK/AXL dual targeting and pan-TAM degraders (KTX-652 and KTX-978). Furthermore, employing primary *ex vivo* dexamethasone-treated bone marrow derived macrophages as well as EGFR-TAM reporter lines, MERTK degraders show significant reduction in surface expression of MERTK and suppress efferocytosis of apoptotic cells to levels that approach Mertk knockout cells, demonstrating functional activity in cell-based assays. *In vivo*, MERTK degraders show dose and time-dependent reduction in MERTK levels in spleen of naïve mice. This approach provides the conceptual rationale for continued development of targeted protein degraders aimed at the TAM family of receptors.

## Methods

### Targeted protein degraders

The compounds reported herein were prepared utilizing the procedures reported in the literature. Ji, N., Mainolfi, N., & Weiss, M. M. (2022). U.S. Patent Application No. 17/258,344.

### Cell culture

EGFR/TAM cell lines were generated and maintained in HAMs/F-12 Medium (Corning) supplemented with 10% (v/v) FBS (Atlanta Biologicals), 100 mg/mL Geneticin (Gold Bio) as previously described (41). EO771 cells were cultured in DMEM medium (Corning) supplemented with 10% (v/v) FBS (Atlanta Biologicals), and 1% Penicillin/Streptomycin. All cells and cell lines were grown at 37 °C in a humidified incubator with 5% CO_2_.

### BMDM and peritoneal macrophage culture

6-8 weeks old C57BL/6 mice were sacrificed and tibias and femurs were collected in PBS as previously described (42, 43). Bones were crushed in mortar and pestle with 5mL IMDM medium supplemented with 10% HI FBS (v/v) and 1% Penicillin/Streptomycin (BMDM medium). Bone marrow cells were resuspended with pipetting and spun down at 1500 rpm for 5 minutes at 4°C. RBC lysis was done with ACK lysis buffer for 5 minutes at room temperature. Cells were strained through 70 μM strainer to remove debris and clumped cells. Strainer was washed with 10 mL BMDM medium and cells were centrifuged at 1500 rpm for 5 minutes to collect the cells. Cell pellet was resuspended in BMDM medium, cells were counted and plated 15 cm tissue culture plates. Culture medium was supplemented with 20 ng/mL of recombinant mouse M-CSF, carrier free (Biolegend). On day 3, half of the medium was replaced with fresh medium containing 20 ng/mL of M-CSF. Differentiated macrophages were ready on day 7 and were detached using scrapers and counted using trypan blue exclusion on hemocytometer. For peritoneal macrophage isolation, 6-8 weeks old C57BL/6 mice were injected intraperitoneally with 1 mL PBS, 48 hours before macrophage isolation. After 48 hours, the peritoneal exudate was collected from mice by washing the peritoneal cavity twice with RPMI medium containing 10% HI FBS and 1% Penicillin/Streptomycin. Total cells in the peritoneal exudate were counted by trypan blue exclusion on hemocytometer and plated in 10 cm tissue culture plates at a density of 10^6^ cells per mL. 24 hours later, the supernatant was replaced with fresh medium, leaving peritoneal macrophages attached to the plate. Macrophages were plated on 12 well tissue culture treated plates at 500,000 cells per well for MERTK degrader treatment and efferocytosis experiments. BMDMs were treated with 0.1 µM Dexamethasone for 18 hours to induce MERTK overexpression. BMDMs not treated with Dexamethasone are referred to as naïve here. For the degrader wash out experiments, 10^6^ differentiated BMDMs were plated on 6 well plates, and treated with degraders or control compounds for 18 hours. 18 hours later, the treatment was removed and cells were washed 3 times with BMDM medium. MERTK was activated by treatment with GAS6 enriched medium and PS liposomes (Avanti polar lipids) at the indicated time points after wash out. For western blot analysis, BMDMs were washed with ice cold PBS at indicated time points, and, lysed and processed as mentioned in the immunoblotting section.

### Efferocytosis assay

Differentiated BMDMs were plated on a 12 well plate, starved in IMDM medium with 0.5% HI FBS, with or without Dexamethasone for 18 hours. Apoptotic cells were prepared by treating Jurkat cells with 1 µM Staurosporine (FUJIFILM). Staurosporine treated cells were incubated for 3 hours in RPMI without serum at 37°C. After 5 hours, apoptotic cells were washed and labelled with 100 ng/mL pHrodo Red (ThermoFisher) for 30 minutes, followed by washing twice with PBS containing 1% BSA and 1 mM EDTA and once with IMDM only. Labelled apoptotic cells were resuspended in IMDM medium containing 10% FBS added to plated macrophages at 3:1 ratio and incubated for 45 minutes at 37°C. Macrophages were washed twice with PBS and detached by scraping. Efferocytosis was analyzed by flow cytometry, measuring the % of pHrodo+ cells within CD11b+ F4/80+ macrophages (42).

### Flow cytometry

BMDM, or peritoneal macrophages were collected after treatment with degraders, or after efferocytosis and washed with cell staining buffer (BD Biosciences). Cells were counted and 500,000 cells were collected in 96 well V bottom plates for staining. Macrophages were incubated with Fc block (1:50) (Biolegend) and incubated at 4°C for 15 minutes. Cells were then stained with conjugated antibodies at 1:100 dilution for 30 minutes at 4°C. Macrophages were stained with anti-CD11b-PercpCy5.5 and anti-F4/80-APC (Biolegend) and for MERTK expression, macrophages were also stained with anti-MERTK-PE (Biolegend). Similarly, EO771 and EGFR/TAM cells were collected, washed with cell staining buffer and stained with anti-AXL-PE (Biolegend) or anti-human EGFR-PE antibodies respectively for 30 minutes at 4°C. After staining, all cells were washed 2 times with cell staining buffer and fixed with 4% paraformaldehyde (PFA) for 15 minutes at room temperature. Cells were washed with flow buffer, transferred into flow tubes and analyzed by BD Fortessa or BD LSR II. Flow cytometry data was analyzed using FlowJo software, version 9.

### Proliferation assay using incucyte

EO771 cells were plated in a 96 well flat bottom plate at 20,000 cells/well in DMEM containing 10% FBS and 1% Penn/Strep. 100 µL of media containing 100 nM degrader was added to the cells in triplicates. Using Sartorius Incucyte SX1 real time imager, phase contrast images were acquired for all wells (4 images per well) every 4 hours, for a total time period of 72 hours. For analysis, % of confluence was calculated for each image and each well within a group and plotted against time.

### Immunoblotting

Whole cell lysates were prepared in HNTG lysis buffer (20mM HEPES, pH 7.5, 150 mM NaCl, 10% Glycerol) supplemented with 10% triton X-100, 1mM PMSF, 1mM sodium orthovanadate, 10mM sodium molybdate, 1mM EDTA and 1% protease inhibitor cocktail (Thermo Fisher). Bradford assay was done to measure protein concentration and 40 µg of protein for each sample was loaded onto a SDS reducing gel (10% acrylamide). SDS gel electrophoresis was carried out at 60 V and then at 120 V when proteins entered the resolving gel. Proteins were transferred from the gel onto a PVDF membrane using the wet transfer method at a constant current of 0.25 A for 70 minutes. Blots were blocked in 5% non-fat dried milk made in TBS for 1 hour at room temperature. Blots were incubated overnight with primary antibodies, anti- mouse MERTK (Rndsystems),(molecular weight 110 kDa), anti-pMERTK (PhosphoSolutions),(molecular weight 180 kDa), and anti-β-ACTIN (Cell signaling Technologies),(molecular weight 75 kDa) at 1:1000 dilution, at 4°C. Next morning, blots were washed thrice with TBST for intervals of 15 minutes. The blots were then incubated with secondary antibodies, anti-mouse IgG-HRP or anti-goat IgG-HRP (1:4000) for 1 hour at room temperature. After 1 hour, the blots were washed thrice with TBST for intervals of 15 minutes. The blots were developed with ECL substrate (BioRad) and chemiluminescent images were captured using Biorad ImageDoc.

### PK/PD in naïve mice

Balb/c female mice were randomized based on weight to study groups (N=3/group). A single subcutaneous (s.c.) injection of KTX-652, or vehicle (10% DMSO, 10% Cremophore EL and 80% “20% Hydroxy-Beta-Cyclodextrin” in saline) was administered subcutaneously at a dosing volume of 5 mL/kg to each treatment group. Final doses tested were 3, 30 and 100 mg/kg. To correlate PK and PD, mice were euthanized following dosing at 15 minutes, 30 minutes, 3 hours, 6 hours, 24 hours, 48 hours, and 72 hours for sample collection. For PK sampling, 20 µL of whole blood was collected via cardiac puncture into 1.5 mL EDTA coated centrifuge tubes and centrifuged at 4000 g for 5 min, at 4°C to separate the plasma. One third of the spleen was also collected and snap frozen in liquid nitrogen. KTX-978 was administered subcutaneously at dose of 10 mg/kg and samples were collected at 0, 0.25, 0.5, 1, 4, 8 and 24 hours. For the PD experiments, KTX-978 or vehicle (10% DMSO, 10% Cremophore EL and 80% “20% Hydroxy-Beta-Cyclodextrin” in saline) was injected subcutaneously in mice bearing CT26 tumors of 250-300 mm^3^ at doses 30 mg/kg and 100 mg/kg. Spleens and tumors were harvested at 6, 24 and 48 hours for pharmacodynamics. For PD sampling, one third of the spleen was snap frozen for western blot analysis of MERTK. All samples were stored at -80°C until further processing.

### Pharmacokinetics sample preparation

The desired serial concentrations of working solutions were achieved by diluting stock solution of analyte with 50% acetonitrile in water solution. 3 µL of working solutions (20, 50, 100, 500, 1000, 5000, 10000 ng/mL) were added to 30 μL of the blank Balb/c mice spleen homogenate to achieve calibration standards of 2~1000 ng/mL (2, 5, 10, 50, 100, 500, 1000 ng/mL) in a total volume of 33 μL. Three quality control samples at 2 ng/mL, 50 ng/mL, and 800 ng/mL for spleen homogenate were prepared independently of those used for the calibration curves. QC samples were prepared on the day of analysis in the same way as calibration standards. 33 μL of standards, 33 μL of QC samples and 33 μL of unknown samples (30 µL of Balb/c mice spleen homogenate with 3 µL of blank solution) were added to 200 μL of acetonitrile containing internal standard mixture for precipitating protein respectively. Then the samples were vortexed for 30 seconds. After centrifugation at 4°C, 4700 rpm for 15 minutes, the supernatant was diluted 3 times with water. 10 µL of diluted supernatant was injected into the LC/MS/MS system for quantitative analysis.

### Pharmacodynamics – immunoblotting (*Ex vivo*)

Frozen spleen and tumor samples were thawed on ice. The samples were homogenized and lysed by adding 500 µL of pre-chilled RIPA lysis buffer with protease and phosphatase inhibitor was added to each sample and one steel ball was added to each tube. The tubes with sample were placed on TissueLyzer for 4 minutes at 30HZ. The samples continued to lyse at 4°C for one hour followed by spinning at 13,000 rpm for 20 minutes. Supernatants were collected and quantified by BCA methods to adjust sample concentration to 4 µg/µL. 100 µL of sample was transferred to a fresh tube containing 25 µL of 5x loading buffer, and then heated to 100°C for 10 minutes. 20 µL was loaded onto SDS-PAGE gel and run at 120V for 1.5 hours. A wet transfer of the gel was performed at 250 mA for 2.5 hours onto a PVDF membrane. The membranes were blocked with LICOR blocking buffer for one hour, and then washed three times with TBST. The primary antibodies were prepared in blocking buffer with 0.1% Tween-20 at 4°C overnight. The goat anti-MERTK (R&D AF591) (molecular weight 110 kDa) was used at 1:500 and mouse anti-beta-actin (Cell signaling technologies #3700) (molecular weight 75 kDa) at 1:10,000. After overnight blocking, membranes were washed three times with TBST. The membranes were incubated with secondary antibody for one hour at room temperature (anti-goat IgG (Licor 926-32214) at 1:5000 and anti-mouse IgG (Licor 926-68070) at 1:5000. Membranes were washed three times with TBST and read on Licor.

### Supplementary methods for selectivity proteomics analysis

Human Jurkat cells were preincubated in RPMI Medium (Gibco™) supplemented with 10% (v/v) FBS (Atlanta Biologicals), and grown at 37°C in a humidified incubator with 5% CO2. Jurkat cells were then treated with DMSO, KTX-652 (20nM) and KTX-978 (20nM), respectively, for 24 hours in triplicates and collected for selectivity proteomics analysis. Cell samples were lysed and digested using EasyPep™ MS Sample Prep Kit (Thermo Scientific™). Tryptic peptides were labeled with 11-plex TMT reagents (Thermo Scientific™) for quantitative analysis, and subject to off-line high-pH reversed-phase fractionation using Agilent 1290 Infinity LC system (Agilent technologies). Twenty-four fractionations were collected and desalted by StageTips (Affinisep) prior to MS analysis.

MS data acquisition was performed on a Q Exactive™ Plus Hybrid Quadrupole-Orbitrap™ Mass Spectrometer coupled with EASY-nLC™ 1200 System (Thermo Fisher Scientific). Briefly, each fractionation was reconstituted in 50 µl 0.1%TFA/H_2_O. Two microliter sample was injected and separated by a reversed phase column packed in-house with 1.8-µm C18 Reprosil-AQ Pur reversed-phase beads (Dr. Maisch GmbH) over 110 min. Full MS scan was acquired using a resolution of 70K at *m/z* 200, an AGC target of 3e6 and a mass range of *m/z* 300-1800. MS/MS spectra were acquired by data-dependent acquisition (DDA) mode. Top 15 precursor ions were selected and fragmented using the parameters below: a resolution of 35K at *m/z* 200, an isolation window of 0.7 *m/z*, HCD collisional energy of 30 and a minimum AGC target of 2.5e3. Spectra were analyzed using MaxQuant (versions 1.6.14.0) and Andromeda. The MS2 spectra were searched against the UniProt Human FASTA Database (version 01/15/2019). Carbamidomethylation of cysteine was set as a fixed modification and N-acetylation and oxidation of methionine as variable modifications. Enzyme specificity was set to trypsin and up to two missed cleavage sites were allowed. False discovery rate (FDR) of 1% was applied to filter identifications at peptide and protein levels. TMT-11 plex was selected as the isobaric label approach within a reporter ion MS2 experiment. Statistical analysis was carried out using the Limma statistical package. A weighted cutoff between statistical significance and fold-change was applied. Proteins with absolute off-target value more than 1 were considered as significantly changed proteins.

## Results

### Targeted protein degraders decrease surface MERTK expression and suppress efferocytosis in primary murine macrophages

**Figure 1:**
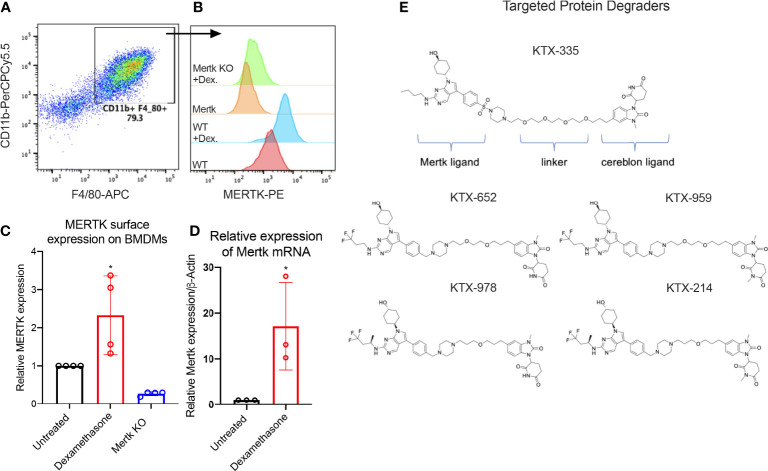
Targeted protein degraders (TPD) for targeting MERTK on murine BMDMs. **(A)** BMDMs are defined as CD11b+ F4/80+ cells by flow cytometry. **(B)** Histograms show that MERTK is expressed on murine BMDMs and is highly induced by treatment with 0.1 µM of dexamethasone, whereas Mertk KO BMDMs do not express MERTK. **(C)** MERTK levels in WT, dexamethasone treated and Mertk KO BMDMs are measured by flow cytometry (Bar graphs represent mean of MFI ± SD, analyzed by ordinary one way ANOVA, n=3 *=p<0.05) and **(D)** mRNA expression is measured by qRTPCR (Bar graphs represent mean relative expression ± SD, analyzed by ordinary one way ANOVA, n=3 *=p<0.05; only statistically significant results have been represented by asterisks (*), for all figures in this paper). **(E)** shows the chemical structures of degraders, KTX-335 consists of a MERTK ligand conjugated to a cereblon ligand by a linker. KTX-959 and KTX-214 are compounds where the cereblon ligand is methylated and unable to engage cereblon, hence they serve as negative controls for KTX-652 and KTX-978, respectively.

Previous studies have shown that MERTK, expressed on bone marrow-derived or on peritoneal elicited macrophages, acts as a pre-eminent receptor for efferocytosis, the process by which apoptotic cells are engulfed by phagocytes (11, 42, 44–46). In this study, BMDMs were obtained by differentiating murine bone marrow monocytes with M-CSF containing media for 7 days. Differentiated BMDMs were defined as cells expressing both CD11b and F4/80 markers as indicated by the flow cytometry plot (**Figure 1A**). MERTK was highly expressed by BMDMs derived from C57BL6/WT mice (indicated as WT BMDMs), in addition to being overexpressed after treatment with the anti-inflammatory corticosteroid, dexamethasone (**Figure 1B**). Dexamethasone mediated increase in MERTK was observed at both the protein level by flow cytometry (**Figure 1C**), as well as at the mRNA level by qRT-PCR (**Figure 1D**). No MERTK expression was noted in macrophages from Mertk knockout mice (Mertk KO), serving as a negative control (**Figures 1B, C**).

To assess the activity of TPDs for MERTK and other TAM family kinases, we designed heterobifunctional degraders composed of two ligands conjugated by a linker, whereby one of the ligands is a tyrosine kinase inhibitor targeting MERTK while the other is a ligand that binds the E3 ubiquitin ligase, cereblon. The MERTK ligand was inspired by work carried out by researchers at the University of North Carolina (UNC) Earp III, H. S. (2018). *U.S. Patent Application No. 15/766,612*., in conjunction with a novel ligand targeting cereblon that lacks any immunomodulatory (IMiD) activity (**Figure 1E**). Ji, N., Mainolfi, N., & Weiss, M. M. (2022). U.S. Patent Application No. 17/258,344.

**Figure 2:**
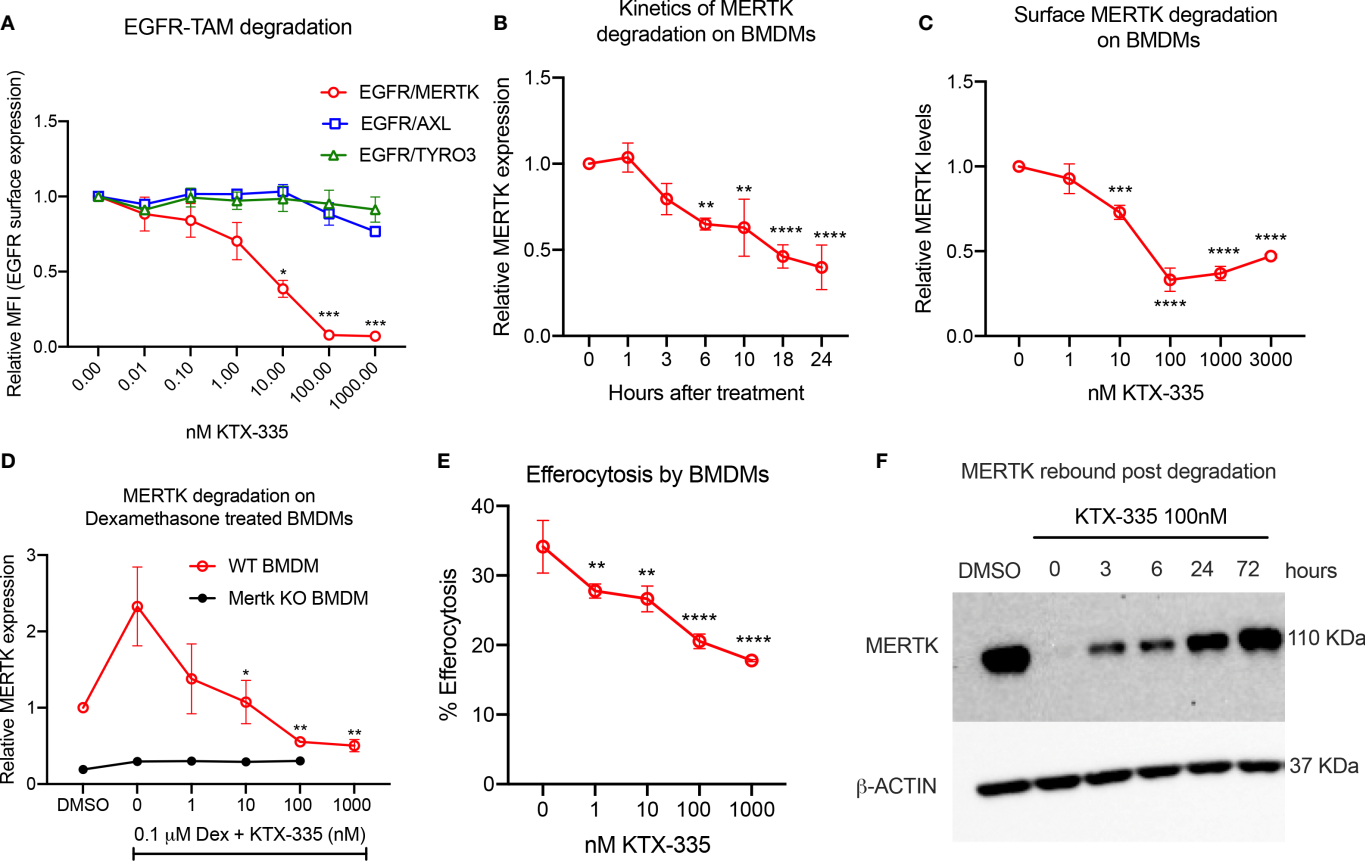
KTX-335 selectively degrades MERTK and suppresses efferocytosis. **(A)** Treatment of EGFR/TAM chimeric cell lines with KTX-335 shows degradation specific for MERTK and not for AXL or TYRO3. (Plot shows relative MFI of EGFR ± SD normalized to untreated, analyzed by two way ANOVA, Dunnett’s multiple comparison test, n=3, *=p<0.05, ** =p < 0.01, *** = p <0.001, **** = p < 0.0001, compared to untreated, also shown as 0 nM). **(B)** shows kinetics of degradation of MERTK on naïve BMDMs treated with KTX-335 for 1, 3, 6, 10, 18 and 24 hours. MERTK expression is measured by flow cytometry using an anti-MERTK-PE antibody and y- axis represents relative MFI (PE) ± SD normalized to the 0 hour time point (Ordinary One way ANOVA, n=4, * =p<0.05, ** =p < 0.01, *** = p <0.001, **** = p < 0.0001, compared to 0 hour time point). **(C)** KTX-335 reduces surface MERTK expression on naïve BMDMs (18 hours treatment) in a dose dependent manner up-to 100 nM, and demonstrating the hook effect at higher concentrations, (Plot shows relative MFI of MERTK-PE ± SD normalized to untreated, analyzed by one-way ANOVA, n=4, **** = p < 0.0001, compared to untreated). **(D)** KTX-335 also efficiently reduces MERTK expression on Dexamethasone induced BMDMs (Plot shows relative MFI of MERTK-PE ± SD normalized to untreated, analyzed by one-way ANOVA, n=4, * =p<0.05, ** =p < 0.01 compared to untreated). **(E)** KTX-335 reduces efferocytosis of apoptotic cells in naïve BMDMs, (Plot shows the mean percentage of pHrodo+ cells ± SD within the CD11b+ F4/80+ gate, analyzed by ordinary one way ANOVA, n=3, * =p<0.05, ** =p < 0.01, *** = p <0.001, **** = p < 0.0001. **(F)** Western blot shows rebound of MERTK in BMDMs post wash out of KTX-335 at 0, 3, 6, 24 and 72 hours.

In order to assess the selectivity of KTX-335 for the targeted degradation of MERTK versus other TAM family members TYRO3 and AXL, we employed EGFR-TAM chimeric cell lines as previously reported by our group (41). EGFR-TAM receptors are engineered to express the extracellular and transmembrane domain of the human EGFR with the intracellular kinase and cytoplasmic tails of the individual TAMs, thus normalizing TAM post-receptor signaling following the common addition of recombinant EGF as an activating ligand (41). As shown in **Figure 2A**, treatment with KTX-335 specifically decreased EGFR-MERTK expression, but not EGFR-TYRO3 or EGFR-AXL, as evident by flow cytometry assessed with an antibody directed to the EGFR ectodomain. To study the kinetics of degradation, we measured surface MERTK on naïve BMDMs using flow cytometry, after treating BMDMs with 100 nM KTX-335 for up to 24 hours of post-exposure (**Figure 2B**). As indicated, MERTK was optimally degraded at 18 to 24 hours post treatment, and therefore the 18-hour time point was used in subsequent assays.

To explore functional utility *in vitro*, we initially tested KTX-335 on primary naïve bone marrow derived macrophages (BMDMs) (**Figure 2C**) or dexamethasone treated BMDMs (**Figure 2D**), the latter elevating MERTK expression for phenotypic analysis (**Figures 1B, C**). As shown in **Figure 2C**, when examined over a range of 1 nM to 3000 nM, KTX-335 reduced MERTK surface expression in a dose-dependent manner. Maximal reduction was 70%, compared to untreated BMDMs, and occurred at treatment with 100 nM KTX-335. At higher concentrations of KTX-335 (3000 nM), the ability to degrade MERTK was partially diminished. This is likely due to the “hook effect” wherein there is a reduction in degradation efficiency that is driven by the increased formation of binary complexes, precluding the formation of a productive ternary complex (47). KTX-335 also effectively reduced surface MERTK on dexamethasone treated WT BMDMs in a dose dependent manner at 1-1000 nM concentrations, showing maximal degradation at 100 nM and 1000 nM (**Figure 2D**). MERTK degradation by KTX-335 (100 and 1000 nM) on dexamethasone treated BMDMs (red) approached the low levels of MERTK expression on Mertk KO BMDMs (black) (**Figure 2D**).

Using a functional assay, KTX-335 dose-dependently (1-1000 nM) decreased efferocytosis of apoptotic Jurkat cells by BMDMs (**Figure 2E**). Further, to determine the rate at which MERTK rebounds after removal of TPDs, BMDMs were treated with 100 nM KTX-335 for 18 hours, after which compounds were washed three times, and cultured with BMDM medium for an additional 0, 3, 6, 24 and 72 hours. As shown in **Figure 2F** MERTK was not resynthesized to pre-treatment levels for at least 24 hours after treatment with KTX-335. These data imply that unlike small molecule drug washout, targeted protein degradation of MERTK permits extended inhibition of activity until proteins are maximally resynthesized and expressed.

### KTX-652 is a MERTK and AXL dual degrader that potently inhibits efferocytosis

**Figure 3:**
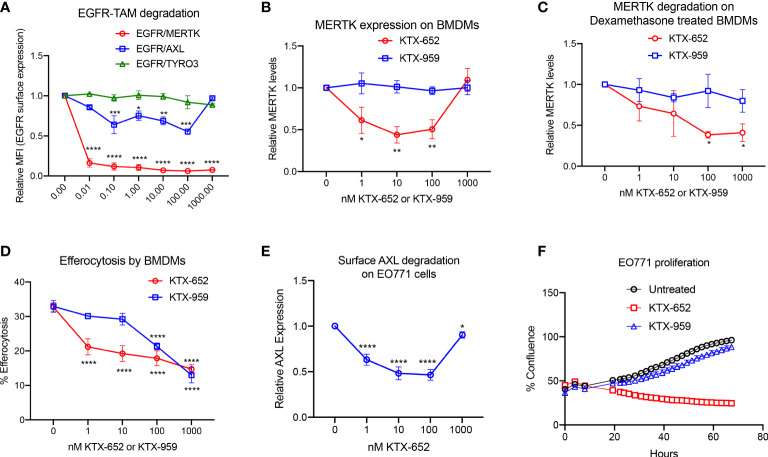
KTX-652 is a MERTK and AXL dual degrader that potently inhibits efferocytosis. **(A)** Flow cytometric assay with EGFR/TAM chimeric cell lines shows that KTX-652 targets both MERTK and AXL (Plot shows relative MFI of EGFR ± SEM normalized to untreated, analyzed by mixed effects analysis, Dunnett’s multiple comparison test, n=5, *=p<0.05, ** =p < 0.01, *** = p <0.001, **** = p < 0.0001, compared to untreated). **(B)** KTX-652, but not KTX-959 reduces MERTK expression on naïve BMDMs (Plot shows MFI ± SD normalized to untreated, analyzed using two way ANOVA and Dunnett’s multiple comparisons test, n=4, * =p<0.05, ** =p < 0.01, *** = p <0.001, **** = p < 0.0001, compared to untreated). **(C)** KTX-652 but not KTX-959 reduces MERTK expression on dexamethasone treated BMDMs (Plot shows MFI ± SD normalized to untreated, two way ANOVA and Dunnett’s multiple comparisons test, n= 4,* =p < 0.05). **(D)** KTX-652 is effective at inhibiting efferocytosis of apoptotic cells at nM concentrations in BMDMs (Plot shows the mean of percentage of pHrodo+ cells ± SD within the CD11b+ F4/80+ gate, analyzed by two way ANOVA, Dunnett’s multiple comparison test, n=3, *=p<0.05, ** =p < 0.01, *** = p <0.001, **** = p < 0.0001). **(E)** KTX-652 degrades surface AXL on EO771 cells, measured by flow cytometry (Plot shows relative MFI ± SD normalized to untreated, analyzed by two way ANOVA and Dunnett’s multiple comparisons test, n= 4, *=p<0.05, ** =p < 0.01, *** = p <0.001, **** = p < 0.0001). **(F)** shows the functional consequence of AXL degradation in EO771 cells, shown by reduced proliferation of EO771 cells treated with 100 nM KTX-652 (Plot depicts % confluence as a function of time, as measured by Incucyte).

In recent years, it has been observed that TYRO3, AXL, and MERTK receptors have complex non-overlapping roles in cancer biology, acting both as oncogenic kinases, and as inhibitory receptors that suppress innate immunity on macrophages and DCs, collectively providing a rationale for the concept of “pan-TAM inhibitors” (17). For example, we have shown that AXL and MERTK have distinct activities using an EO771 breast cancer model, whereby AXL drives tumor progression and proliferation on tumor cells while MERTK drives immune escape on macrophages in the tumor microenvironment (42). KTX-652 (shown in **Figure 1E**) is an example of a heterobifunctional molecule that demonstrates more potent degradation of MERTK, while also targeting AXL. To assess the specificity of KTX-652 on the TAM family, we first tested KTX-652 on EGFR-TAM chimeric cell lines whereby this TPD was more promiscuous than KTX-335, capable of degrading MERTK and AXL to a lesser extent, but not TYRO3 (**Figure 3A**). KTX-652 was also more potent compared to KTX-335, and showed a maximum MERTK degradation of 60% at 10 nM in naïve BMDMs, (**Figure 3B**) and 100 nM in dexamethasone treated BMDMs (**Figure 3C**). Efferocytosis of apoptotic Jurkat cells by BMDMs was inhibited at even a low concentration of 1 nM (**Figure 3D**). KTX-959, an N-methylated analog of KTX-652 which lacks the ability to bind cereblon, was synthesized and used as a negative control for degradation. As expected, KTX-959 was unable to degrade MERTK on either naïve or dexamethasone treated BMDMs (**Figures 3B, C**), and was as effective, but less potent in the efferocytosis assay than KTX-652 (**Figure 3D**). To test the ability of KTX-652 to degrade endogenous AXL, we used EO771 cells, which is a luminal B murine mammary cancer cell line that highly expresses surface AXL. As indicated in **Figure 3E**, KTX-652 efficiently reduced AXL on EO771 cells showing maximum reduction of 50% at 10 and 100 nM, as measured by flow cytometry. Like KTX-335, KTX-652 also showed an apparent hook effect, with reduced efficiency at the highest concentration of 1000 nM (**Figures 3B, E**) Since AXL is characterized as an oncogenic RTK, and a driver of cancer cell survival and proliferation, we assessed proliferation of EO771 upon KTX-652 mediated Axl degradation, and as indicated, KTX-652 significantly reduced their proliferation and survival, (**Figure 3F**). These data support the potential to dually target MERTK on macrophages and AXL on tumor cells as a therapeutic anti- cancer strategy.

### A single treatment of KTX-652 caused dose and time-dependent MERTK degradation in mouse spleen

**Figure 4:**
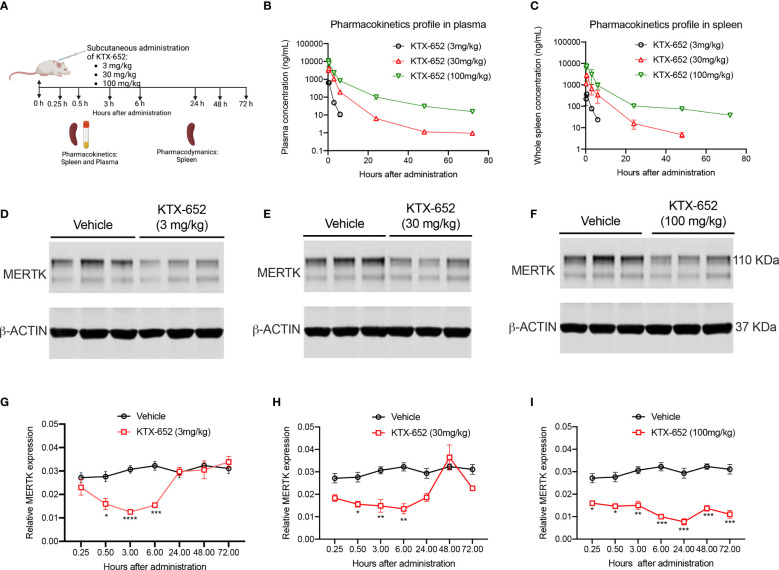
A single dose of KTX-652 causes dose and time-dependent MERTK degradation in mouse spleen. **(A)** Schematic showing workflow of PK and PD experiments. Single dose of KTX-652 was administered subcutaneously at 3 mg/kg, 30 mg/kg or 100 mg/kg. Plasma and spleens were collected at 0.25, 0.5, 3, 6, 24, 48, and 72 hours for PK and PD analysis. Pharmacokinetic profile in spleen **(B)** and plasma **(C)** show good exposure levels up to 72 hours for all 3 concentrations (Plots show mean ± SEM concentrations of KTX-652 in plasma and spleen, 3 mice per group). Western blot representation of MERTK reduction in spleen levels in spleen at 3 hours after administration of KTX-652 at doses, 3 mg/kg **(D)**, 30 mg/kg **(E)** and 100 mg/kg **(F)**. Reduction in MERTK levels is seen up to 6 hours, and increases at 24 hours for 3 mg/kg dose **(D, G)**, and at 48 hours for 30 mg/kg dose **(H)**. MERTK levels remain low for 72 hours at 100 mg/kg **(I)**. (Quantification of western blots, represented by relative MERTK/B-ACTIN, analyzed by Sidak’s multiple comparisons test, * =p<0.05, ** =p < 0.01, *** = p <0.001, **** = p < 0.0001, 3 mice per group, per time point.

To extend *in vitro* cell culture observations and explore whether KTX-652 can target and degrade MERTK *in vivo*, KTX-652 was administered subcutaneously into naïve Balb/c mice at 3 doses, 3 mg/kg, 30 mg/kg, and 100 mg/kg as outlined in **Figure 4A**. Exposure of KTX-652 in both spleen (**Figure 4B**) and plasma (**Figure 4C**) were assessed at 0.25, 0.5, 3, 6, 24, 48, and 72 hours post injection. At 100 mg/kg both spleen and plasma KTX-652 concentrations were maintained above 100 ng/mL for 48 hours. MERTK expression in spleen at the 3-hour time point(s) showed significant reduction at all doses compared to vehicle (n=3; **Figures 4D–F**) Western blots for all doses at all time points are shown in **Supplemental Figure 2**, showing similar trends and reproducibility. The rebound of MERTK after a single dose is illustrated in **Figures 4G–I**. MERTK levels began to increase at the 3 mg/kg dose (**Figure 4G**) and 30 mg/kg, (**Figure 4H**) between 6 hours and 24 hours, while degradation was maintained for up to 72 hours at 100 mg/kg (**Figure 4I**).

### KTX-978 functions as a pan-TAM degrader

**Figure 5:**
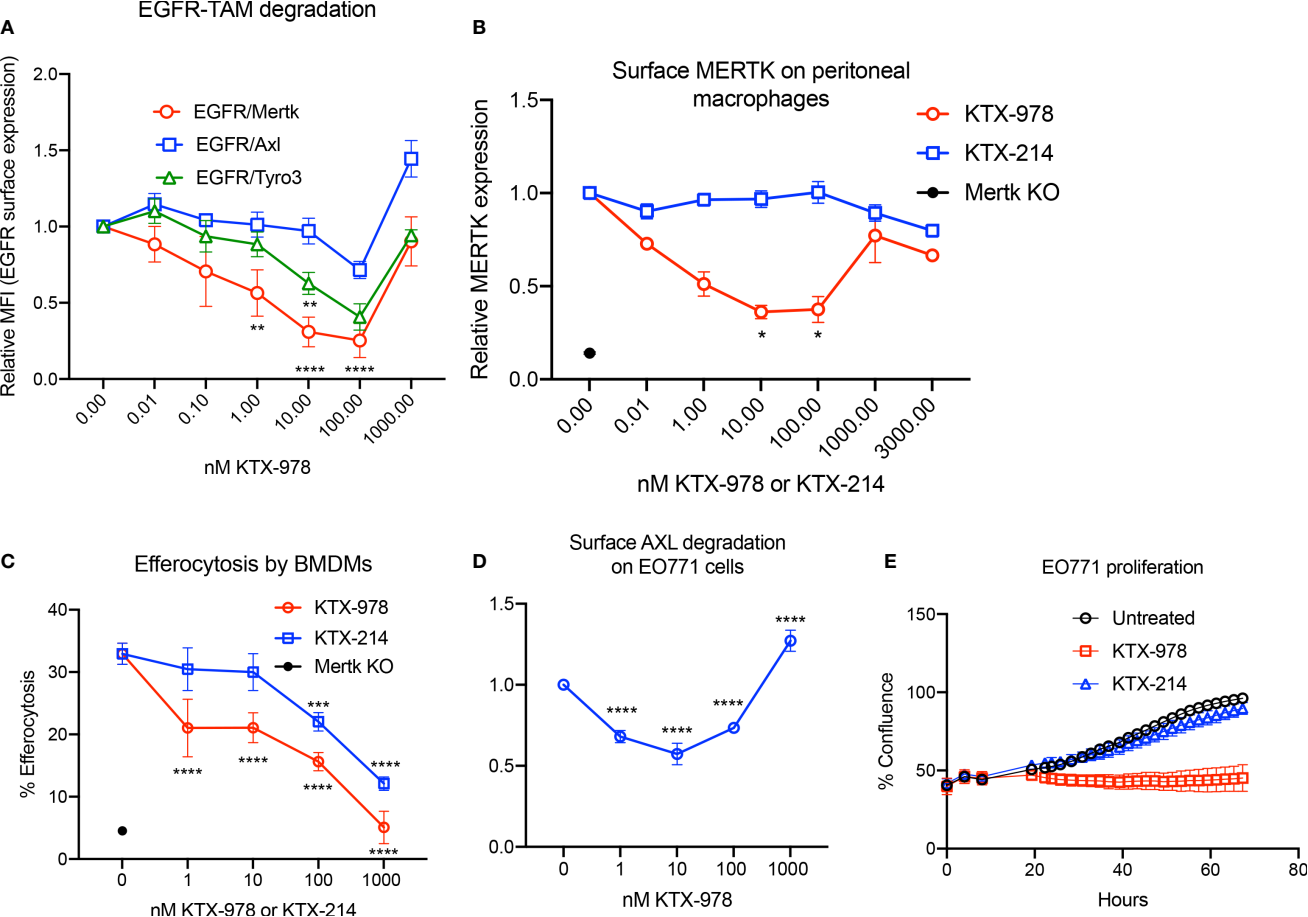
KTX-978 functions as a pan-TAM degrader **(A)** Treatment on EGFR/TAM chimeric cell lines shows that KTX-978 is a pan-TAM degrader (Plot shows relative MFI of EGFR ± SEM normalized to untreated, analyzed by mixed effects analysis, Bonferroni’s multiple comparison test, n=3, *=p <0.05, ** =p < 0.01, *** = p < 0.001, **** = p < 0.0001, compared to untreated). **(B)** KTX-978 induces targeted degradation of MERTK on peritoneal macrophages from C57BL/6 mice, while KTX-214 has a reduced effect. (Plot represents relative MFI ± SD normalized to untreated, analyzed by two way ANOVA, Dunnett’s multiple comparison test, n=3, *=p<0.05, ** =p < 0.01, *** = p <0.001, **** = p < 0.0001). **(C)** Similarly, KTX-978 and KTX-214 with lesser potency, reduces apoptotic cell efferocytosis in BMDMs (Plot shows the mean of percentage of pHrodo+ cells ± SD within the CD11b+ F4/80+ gate, analyzed by Sidak’s multiple comparisons test, n=3 *=p<0.05, ** =p < 0.01, *** = p <0.001, **** = p < 0.0001). **(D)** KTX-978 degrades surface AXL on EO771 cells, up to 100 nM measured by flow cytometry (Plot shows relative MFI ± SD normalized to untreated, analyzed by two way ANOVA and Dunnett’s multiple comparisons test, n= 3, * =p<0.05, ** =p < 0.01, *** = p <0.001, **** = p < 0.0001). **(E)** shows the functional consequence of AXL degradation in EO771 cells, shown by reduced proliferation of EO771 cells treated with 100 nM KTX-978 (Plot depicts % confluence as a function of time, as measured by Incucyte).

Heterobifunctional molecules capable of showing pan-TAM degradation were also identified (shown in **Figure 1E**). As indicated in **Figure 5A**, KTX-978, demonstrated pan-TAM degradation in the EGFR-TAM flow cytometry-based reporter assay. KTX-978 had activity against all three receptors, with maximum degradation of MERTK (66%), AXL (25%) and TYRO3 (55%) observed at 100 nM (with AXL having the weakest maximum degradation). KTX-214, the N-methylated analog of KTX-978 that lacks cereblon engagement, was used as a negative control for degradation and as expected showed minimal to no impact on MERTK degradation on the EGFR-MERTK cell line (**Supplemental Figures 3A, B**). Degradation of EGFR-MERTK by 100 nM KTX-978 (compared to 100 nM KTX-214) also reduced downstream activation of MERTK by EGF activation, as measured by p-MERTK levels on Western blot (**Supplemental Figure 3C**). MERTK activation in BMDMs by GAS6 and PS liposomes (shown by p-MERTK) was reduced at 6 and even 12 hours after washing out KTX-978, whereas MERTK activation was restored after washing out KTX-214 at same time points, showing that MERTK levels remain low sustainably after removal of degrader KTX-978 (**Supplementary Figure 3D**). KTX-978 reduced MERTK expression on murine peritoneal macrophages, showing maximal degradation of 65% at 10 nM. (To levels approaching MERTK KO levels) (**Figure 5B**). KTX-978 more prolifically decreased efferocytosis in BMDMs compared to KTX-214. (**Figure 5C**). KTX-978 also degraded endogenous AXL on EO771 cells, showing maximal degradation of 50% at 10 nM, measured by reduction of surface AXL by flow cytometry (**Figure 5D**). Finally, analogous to the studies in **Figure 3F**, proliferation of EO771 cells was also strongly inhibited by the degradation of AXL by KTX-978, but not by KTX-214 (**Figure 5E**). The ability of KTX-978 and KTX-652 to degrade AXL on EO771 cancer cells and inhibit their proliferation further supports the utility of these pan-TAM TPDs in anti-cancer therapy.

To explore their degradation selectivity beyond TAM receptors, KTX-652 and KTX-978 induced changes in protein abundance were assessed by mass spectrometry-based quantitative proteomics. KTX-978 shows no off targets and none of the observed off-targets for KTX-652 are reported to impact TAM biology or efferocytosis (**Supplemental Figure 1A**). The depth of proteome achieved in this selectivity analysis provides assessment of degrader selectivity against ~8000 human proteins. However, the three TAM proteins remained undetected at this proteome depth. This lack of detection corroborates with generally lower yet functional gene expression levels of TAM receptors in Jurkat cells as observed in the CCLE cell line RNA and protein expression database (https://depmap.org/portal/). As noted in **Supplementary Figures 1B, C and D**, AXL & MERTK are generally very low or low in abundance while TYRO3 remained undetected in ~70% of CCLE cell lines despite medium transcript levels. In contrast with TYRO3, CRBN (CEREBLON) at comparable transcripts level shows protein detection each time an RNA signal is seen, suggesting post transcriptional regulation of TYRO3 expression and hence lack of detection at proteomics sensitivity in CCLE as well as current study.

### KTX-978 demonstrates potent degradation of MERTK in spleen and tumor

**Figure 6:**
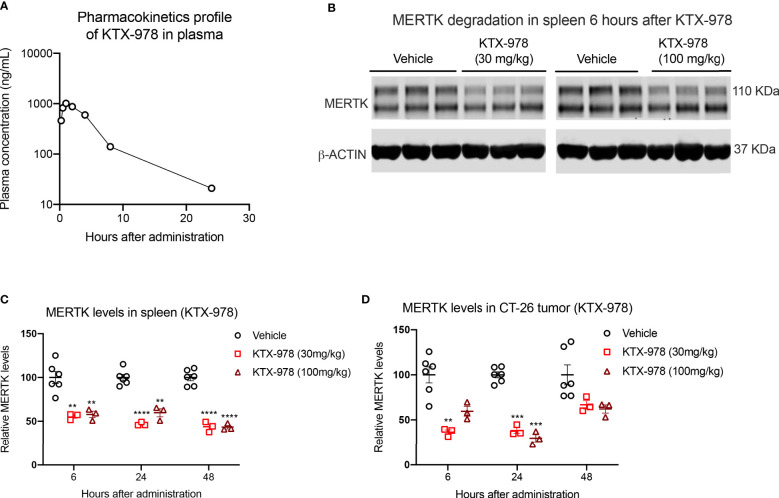
KTX-978 is a potent degrader of MERTK in spleen and tumor. **(A)** Plasma concentration of KTX-978 was maintained above 10 ng/mL following a single subcutaneous dose of 10 mg/kg up to 72 hours. **(B)** MERTK degradation in spleen after 6 hours of treatment with 30 mg/kg and 100 mg/kg dose shown by western blotting. Pharmacodynamic profile showing sustained degradation of MERTK in spleen at doses 30 mg/kg and 100 mg/kg after 6, 24 and 48 hours. **(C)** and in the CT-26 tumor at doses 30 mg/kg and 100 mg/kg for 24 hours **(D)**. (Quantification of western blots, represented by relative MERTK/B-ACTIN, analyzed by Sidak’s multiple comparison’s test, n=9, ** =p < 0.01, *** = p <0.001, **** = p < 0.0001).

Following the observations that KTX-978 is a potent pan-TAM degrader, we assessed pharmacokinetic and pharmacodynamic properties *in vivo*. Analogous to KTX-652, plasma concentration of KTX-978 could be maintained above 100 ng/mL for up to 8 hours and above 10 ng/mL for up-to 24 hours when administered subcutaneously at a dose of 10 mg/kg. (**Figure 6A**). Exposure levels of KTX-978 and MERTK protein expression were evaluated in mouse spleen following a single s.c. injection of 30 mg/kg and 100 mg/kg. MERTK showed significant (~50%) degradation at both doses as shown by Western blot images for 6-hour time point (**Figure 6B**). and was sustained up to 24 and 48 hours after injection, as quantified in **Figure 6C**. Since MERTK has been implicated to have oncogenic and pro-tumorigenic functions, we also assessed the capacity of KTX-978 to target MERTK in an orthotopic CT-26 tumor (**Figure 6D**). KTX-978 significantly reduced MERTK levels in CT-26 tumor at 30mg/kg at 6 hours, and at both 30 mg/kg and 100 mg/kg for 24 hours. Collectively, our data support the utility of TPDs for MERTK and TAM kinases for future experimental and therapeutic development.

## Discussion

TAM receptors (TYRO3, AXL, and MERTK), broadly expressed on both tumors cells (as oncogenic kinases) and on myeloid cells (as modulatory regulators that dampen innate immune responses) have gained traction in cancer biology in recent years as therapeutic modalities. Here we report proof-of-concept, first in class TPDs that target MERTK for ubiquitination and targeted protein degradation. Unlike tyrosine kinase inhibitors, or inhibitory monoclonal antibodies that transiently inhibit MERTK (i.e TKIs) or transport MERTK from the cell surface to an intracellular compartment (i.e mAbs), TPDs are expected to eliminate MERTK expression to more robustly phenocopy loss of function knockout mutation. As such, the molecules and strategies outlined in the present study should be adaptable to investigate *in vitro* signaling and functional studies or expanded for *in vivo* therapeutic utility. Moreover, we demonstrate that heterobifunctional degraders can be designed to degrade all TAMs, MERTK and AXL as bifunctional agents, or MERTK alone as a monospecific agent (KTX-335). Together, this targeted protein degradation approach reported herein provides utility for both experimental and mechanistic studies as well as for therapeutic development for cancer and autoimmune/fibrotic disorders where TAM receptors assume pathophysiological functions.

The present strategy employing MERTK and TAM TPDs to modulate protein levels also provides an alternative and complementary strategy to phenocopy Mertk and TAM genetic functional knockout models. For example, recent observations by Akalu et al. (48) showed that an original Mertk mouse knockout generated by homologous recombination in embryonic C57Bl/6 stem cells and backcrossed to B6 mice to obtain Mertk (-/-) mice (49), contains concomitant loss of Tyro3. In these studies, authors showed functionally that a combined phenotype of MERTK and TYRO3 was required for systemic defects in retinal degeneration and decline in photoreceptors in the eye, as well as anti-tumor responses in immune-oncology applications. As such, MERTK TPDs and/or MERTK TPDs that target one or more other TAMs could be assessed as pharmacological agents to mimic genetic TAM knockout phenotypes. This may be particularly attractive to assess true loss of function activities since, as noted above, small molecule kinase inhibitors, via transient inhibition of kinase activity, can inadvertently lead to receptor up-regulation and stabilization following washout. As noted above and supporting this idea, Lauter and colleagues noted that BMS-777607, a pan TAM inhibitor, induced upregulation of AXL cell surface accumulation independent of transcriptional and translational regulation (40), implying that AXL kinase inhibition can inadvertently lead to compensatory receptor upregulation. Moreover, mAbs can lead to receptor dimerization and internalization, and activation of kinases during internalization as illustrated by antagonistic antibodies described by Tavazoie and colleagues (50). Hence, TAM TPDs likely offer versatility to complement knockout studies and particularly assess combined loss of function TAM models.

In addition to the above-mentioned mechanistic studies, MERTK and TAM TPDs are also likely to have therapeutic utility in circumstances where TAM specific, or multi- or pan-TAM inhibitors require empirical determination or where TAM inhibitors require combination with other checkpoint inhibitors such as anti-PD1, anti-CTLA4, or emerging checkpoint targets. In addition to the aforementioned strategies to directly inhibit oncogenic TAM kinases on tumor cells, MERTK on tumor associated M2 macrophages behaves as a preeminent receptor for efferocytosis (45), the latter event is associated with the production of anti-inflammatory cytokines such as IL-10 and TGF-β (51), as well as the production of prostaglandins factors and suppressive long chain fatty acid derive lipids and specialized pro-resolving mediators (52, 53). Indeed, the inhibition of dexamethasone-elicited macrophages is consistent with classical studies by Zizzo and colleagues showing that MERTK is expressed on M2c macrophages (54, 55), a highly efferocytic and tolerogenic subset associated with IL-10 and GAS6 production, factors associated with cancer immune evasion.

Indeed, consistent with this latter idea stemming from MERTK in the fields of autoimmunity and fibrosis (56,) in oncology studies, inhibition of MERTK on tumor associated macrophages blocks efferocytosis, and improves host anti-tumor immunity in several models including an EO771 breast cancer model (42, 43) and an MC38 colon cancer model (57). Inhibition of MERTK on tumor associated macrophages is associated with improved host T cell immunity MERTK inhibition synergizes with anti-PD1 therapeutics, and also show abscopal effects when rechallenged with subsequent tumor burden (42, 57, 58). MERTK is also associated with tissue fibrosis and accumulation of extracellular matrix and stromal elements in response to chronic inflammation and tissue injury, whereby has an important role during tissue remodeling in fibrotic diseases. MERTK is also implicated in Idiopathic Pulmonary Fibrosis (IPF), a fibrotic disease leading to progressive loss of lung function (59). IPF tissues express elevated levels of MERTK and GAS6, and small molecule inhibitors targeting the TAM receptors’ kinase activity exhibited improved anti-fibrotic activity.

Collectively, in the present study, we provide proof of concept feasibility for the use of heterobifunctional degraders that can either selectively target MERTK or can more broadly serve as pan-TAM degraders. MERTK/TAM TPDs show utility both *in vitro* and *in vivo*, demonstrating degradation of surface MERTK on BMDMs and loss of function by reduced efferocytosis of apoptotic cells for all three degraders. While KTX-335 was specific to MERTK, KTX-652 had MERTK and AXL dual specificity and KTX-978 was a pan-TAM degrader. These data corroborate previous studies from our group showing additive anti-tumor responses when AXL KO is combined with MERTK blockade by an anti-MERTK antibody (42). In this case, a single TPD could play multifunctional roles by degrading AXL on tumor cells and inhibiting their proliferation and MERTK on macrophages to induce an anti-tumor immune response. *In vivo* studies showed a good PK-PD correlation for KTX-652 and KTX-978. Collectively, these data support the utility of TPDs for MERTK and TAM kinases for future experimental and therapeutic development.

## Data availability statement

The original contributions presented in the study are included in the article/**Supplementary Material**. Further inquiries can be directed to the corresponding authors.

## Ethics statement

The animal study was reviewed and approved by Rutgers IACUC, Commission on Life Sciences, National Research Council, Standard Operating Procedures (SOPs) of Pharmaron, Inc.

## Author contributions

VG collected data and wrote paper. VD collected data. RB designed experiments and wrote paper. All authors contributed to the article and approved the submitted version.

## Supplementary material

Supplementary Figure 1
**(A)** Proteomic degradation profiles of KT-652 and KT-978 showed that multiple kinases including Aurora Kinase A, CDK6 (Cyclin Dependent Kinase1), AAK1 (Adaptor Associated protein Kinase 1), GAK and SIK2 (Salt Inducible Kinase) were targeted by KT-652, whereas these off- target effects were absent for KT-978. This proteomics analysis assesses selectivity of our degrader compounds against ~8,000 human proteins. However, MERTK, AXL and TYRO3 remained undetected due to their low abundance **(B)** A histogram of Jurkat transcriptome based on CCLE data showing transcript levels (noted in parentheses) for TAM family members and CRBN. **(C)** A scatterplot comparing RNA and protein levels for TAMs & CRBN across CCLE lines. Correlation values are noted in blue. **(D)** A tabulated gene expression summary of TAMs & CRBN across CCLE lines based on DepMap RNA and protein data.

Supplementary Figure 2 Western blot images representation of MERTK degradation in spleens of mice treated with KTX-652 at 3 mg/kg, 30 mg/kg and 100 mg/kg doses, at different time points, 0.25 hours **(A)**, 0.5 hours, **(B)**, 6 hours **(C)**, 24 hours **(D)**, 48 hours **(E)**, and 72 hours **(F)**.

Supplementary Figure 3 Flow cytometry histograms show that KTX-978 **(A)** degrades MERTK on EGFR-MERTK chimeric cell lines at nanomolar concentrations, whereas KTX-214 **(B)** demonstrates no degradation. Effect of KTX-978 is further shown by reduced downstream activation of p-MERTK when EGFR-MERTK chimeric cell lines are activated by recombinant h-EGF **(C)**. **(D)** MERTK activation on BMDMs is shown by p-MERTK. Total MERTK does not rebound after 6 or 12 hours of removal of KTX-978 and is reflected in reduced p-MERTK activation.

## References

[1] Lemmon MA Schlessinger J. Cell signaling by receptor tyrosine kinases. Cell (2010) 141(7):1117–34. doi: 10.1016/j.cell.2010.06.011 PMC2914105 20602996

[2] Schlessinger J. Receptor tyrosine kinases: legacy of the first two decades. Cold Spring Harb Perspect Biol (2014) 6(3). doi: 10.1101/cshperspect.a008912 PMC3949355 24591517

[3] Snuderl M Fazlollahi L Le LP Nitta M Zhelyazkova BH Davidson CJ. Mosaic amplification of multiple receptor tyrosine kinase genes in glioblastoma. Cancer Cell (2011) 20(6):810–7. doi: 10.1016/j.ccr.2011.11.005 22137795

[4] Yamaoka T Ohba M Ohmori T. Molecular-targeted therapies for epidermal growth factor receptor and its resistance mechanisms. Int J Mol Sci (2017) 18(11). doi: 10.3390/ijms18112420 PMC5713388 29140271

[5] Zwick E Bange J Ullrich A. Receptor tyrosine kinase signalling as a target for cancer intervention strategies. Endocr Relat Cancer (2001) 8(3):161–73. doi: 10.1677/erc.0.0080161 11566607

[6] Davra V Kimani SG Calianese D Birge RB. Ligand activation of TAM family receptors-implications for tumor biology and therapeutic response. Cancers (Basel) (2016) 8(12). doi: 10.3390/cancers8120107 PMC5187505 27916840

[7] Lemke G. Biology of the TAM receptors. Cold Spring Harb Perspect Biol (2013) 5(11):a009076. doi: 10.1101/cshperspect.a009076 24186067 PMC3809585

[8] Nguyen KQ Tsou WI Kotenko S Birge RB. TAM receptors in apoptotic cell clearance, autoimmunity, and cancer. Autoimmunity (2013) 46(5):294–7. doi: 10.3109/08916934.2013.794515 23662598

[9] Burstyn-Cohen T. TAM receptor signaling in development. Int J Dev Biol (2017) 61(3-4-5):215–24. doi: 10.1387/ijdb.160285tb 28621419

[10] Lu Q Lemke G. Homeostatic regulation of the immune system by receptor tyrosine kinases of the tyro 3 family. Science (2001) 293(5528):306–11. doi: 10.1126/science.1061663 11452127

[11] Scott RS McMahon EJ Pop SM Reap EA Caricchio R Cohen PL. Phagocytosis and clearance of apoptotic cells is mediated by MER. Nature (2001) 411(6834):207–11. doi: 10.1038/35075603 11346799

[12] Gadiyar V Lahey KC Calianese D Devoe C Mehta D Bono K. Cell death in the tumor microenvironment: implications for cancer immunotherapy. Cells (2020) 9(10). doi: 10.3390/cells9102207 PMC7599747 33003477

[13] Linger RM Keating AK Earp HS Graham DK. TAM receptor tyrosine kinases: biologic functions, signaling, and potential therapeutic targeting in human cancer. Adv Cancer Res (2008) 100:35–83. doi: 10.1016/S0065-230X(08)00002-X 18620092 PMC3133732

[14] Linger RM Keating AK Earp HS Graham DK. Taking aim at mer and axl receptor tyrosine kinases as novel therapeutic targets in solid tumors. Expert Opin Ther Targets (2010) 14(10):1073–90. doi: 10.1517/14728222.2010.515980 PMC3342018 20809868

[15] Engelsen AST Lotsberg ML Abou Khouzam R Thiery JP Lorens JB Chouaib S. Dissecting the role of AXL in cancer immune escape and resistance to immune checkpoint inhibition. Front Immunol (2022) 13:869676. doi: 10.3389/fimmu.2022.869676 35572601 PMC9092944

[16] Graham DK DeRyckere D Davies KD Earp HS. The TAM family: phosphatidylserine sensing receptor tyrosine kinases gone awry in cancer. Nat Rev Cancer (2014) 14(12):769–85. doi: 10.1038/nrc3847 25568918

[17] Lahey KC Gadiyar V Hill A Desind S Wang Z Davra V. Mertk: an emerging target in cancer biology and immuno-oncology. Int Rev Cell Mol Biol (2022) 368:35–59. doi: 10.1016/bs.ircmb.2022.04.004 35636929 PMC9994207

[18] Lemke G Rothlin CV. Immunobiology of the TAM receptors. Nat Rev Immunol (2008) 8(5):327–36. doi: 10.1038/nri2303 PMC2856445 18421305

[19] Mikolajczyk A Mitula F Popiel D Kaminska B Wieczorek M Pieczykolan J. Two-front war on cancer-targeting TAM receptors in solid tumour therapy. Cancers (Basel) (2022) 14(10). doi: 10.3390/cancers14102488 PMC9140196 35626092

[20] Myers KV Amend SR Pienta KJ. Targeting Tyro3, axl and MerTK (TAM receptors): implications for macrophages in the tumor microenvironment. Mol Cancer (2019) 18(1):94. doi: 10.1186/s12943-019-1022-2 31088471 PMC6515593

[21] Sang YB Kim JH Kim CG Hong MH Kim HR Cho BC. The development of AXL inhibitors in lung cancer: recent progress and challenges. Front Oncol (2022) 12:811247. doi: 10.3389/fonc.2022.811247 35311091 PMC8927964

[22] Shen Y Chen X He J Liao D Zu X. Axl inhibitors as novel cancer therapeutic agents. Life Sci (2018) 198:99–111. doi: 10.1016/j.lfs.2018.02.033 29496493

[23] Vouri M Hafizi S. TAM receptor tyrosine kinases in cancer drug resistance. Cancer Res (2017) 77(11):2775–8. doi: 10.1158/0008-5472.CAN-16-2675 28526769

[24] Wu G Ma Z Cheng Y Hu W Deng C Jiang S. Targeting Gas6/TAM in cancer cells and tumor microenvironment. Mol Cancer (2018) 17(1):20. doi: 10.1186/s12943-018-0769-1 29386018 PMC5793417

[25] Chen TJ Mydel P Benedyk-Machaczka M Kaminska M Kalucka U Blo M. AXL targeting by a specific small molecule or monoclonal antibody inhibits renal cell carcinoma progression in an orthotopic mice model. Physiol Rep (2021) 9(23):e15140. doi: 10.14814/phy2.15140 34877810 PMC8652404

[26] Lotsberg ML Wnuk-Lipinska K Terry S Tan TZ Lu N Trachsel-Moncho L. AXL targeting abrogates autophagic flux and induces immunogenic cell death in drug-resistant cancer cells. J Thorac Oncol (2020) 15(6):973–99. doi: 10.1016/j.jtho.2020.01.015 PMC7397559 32018052

[27] Ludwig KF Du W Sorrelle NB Wnuk-Lipinska K Topalovski M Toombs JE. Small-molecule inhibition of axl targets tumor immune suppression and enhances chemotherapy in pancreatic cancer. Cancer Res (2018) 78(1):246–55. doi: 10.1158/0008-5472.CAN-17-1973 PMC5754222 29180468

[28] Li H Liu Z Liu L Zhang H Han C Girard L. AXL targeting restores PD-1 blockade sensitivity of STK11/LKB1 mutant NSCLC through expansion of TCF1(+) CD8 T cells. Cell Rep Med (2022) 3(3):100554. doi: 10.1016/j.xcrm.2022.100554 35492873 PMC9040166

[29] Okura N Nishioka N Yamada T Taniguchi H Tanimura K Katayama Y. ONO-7475, a novel AXL inhibitor, suppresses the adaptive resistance to initial EGFR-TKI treatment in EGFR-mutated non-small cell lung cancer. Clin Cancer Res (2020) 26(9):2244–56. doi: 10.1158/1078-0432.CCR-19-2321 31953310

[30] Peters S Paz-Ares L Herbst RS Reck M. Addressing CPI resistance in NSCLC: targeting TAM receptors to modulate the tumor microenvironment and future prospects. J Immunother Cancer (2022) 10(7). doi: 10.1136/jitc-2022-004863 PMC9305809 35858709

[31] Rios-Doria J Favata M Lasky K Feldman P Lo Y Yang G. A potent and selective dual inhibitor of AXL and MERTK possesses both immunomodulatory and tumor-targeted activity. Front Oncol (2020) 10:598477. doi: 10.3389/fonc.2020.598477 33425754 PMC7793849

[32] Levis M. Quizartinib for the treatment of FLT3/ITD acute myeloid leukemia. Future Oncol (2014) 10(9):1571–9. doi: 10.2217/fon.14.105 PMC6081740 25145428

[33] Minson KA Smith CC DeRyckere D Libbrecht C Lee-Sherick AB Huey MG. The MERTK/FLT3 inhibitor MRX-2843 overcomes resistance-conferring FLT3 mutations in acute myeloid leukemia. JCI Insight (2016) 1(3):e85630. doi: 10.1172/jci.insight.85630 27158668 PMC4855528

[34] Hu X Zheng X Yang S Wang L Hao X Cui X. First-in-human phase I study of BPI-9016M, a dual MET/Axl inhibitor, in patients with non-small cell lung cancer. J Hematol Oncol (2020) 13(1):6. doi: 10.1186/s13045-019-0834-2 31948451 PMC6966871

[35] Jeon Y Kang H Yang Y Park D Choi B Kim J. A novel selective Axl/Mer/CSF1R kinase inhibitor as a cancer immunotherapeutic agent targeting both immune and tumor cells in the tumor microenvironment. Cancers (Basel) (2022) 14(19). doi: 10.3390/cancers14194821 PMC9563311 36230744

[36] Yokoyama Y Lew ED Seelige R Tindall EA Walsh C Fagan PC. Immuno-oncological efficacy of RXDX-106, a novel TAM (TYRO3, AXL, MER) family small-molecule kinase inhibitor. Cancer Res (2019) 79(8):1996–2008. doi: 10.1158/0008-5472.CAN-18-2022 30723115

[37] Kasikara C Kumar S Kimani S Tsou WI Geng K Davra V. Phosphatidylserine sensing by TAM receptors regulates AKT-dependent chemoresistance and PD-L1 expression. Mol Cancer Res (2017) 15(6):753–64. doi: 10.1158/1541-7786.MCR-16-0350 PMC8363069 28184013

[38] Sang J Acquaviva J Friedland JC Smith DL Sequeira M Zhang C. Targeted inhibition of the molecular chaperone Hsp90 overcomes ALK inhibitor resistance in non-small cell lung cancer. Cancer Discovery (2013) 3(4):430–43. doi: 10.1158/2159-8290.CD-12-0440 PMC4086149 23533265

[39] Lovly CM Shaw AT. Molecular pathways: resistance to kinase inhibitors and implications for therapeutic strategies. Clin Cancer Res (2014) 20(9):2249–56. doi: 10.1158/1078-0432.CCR-13-1610 PMC4029617 24789032

[40] Lauter M Weber A Torka R. Targeting of the AXL receptor tyrosine kinase by small molecule inhibitor leads to AXL cell surface accumulation by impairing the ubiquitin-dependent receptor degradation. Cell Commun Signal (2019) 17(1):59. doi: 10.1186/s12964-019-0377-8 31171001 PMC6555758

[41] Kimani SG Kumar S Davra V Chang YJ Kasikara C Geng K. Normalization of TAM post-receptor signaling reveals a cell invasive signature for axl tyrosine kinase. Cell Commun Signal (2016) 14(1):19. doi: 10.1186/s12964-016-0142-1 27595981 PMC5011882

[42] Davra V Kumar S Geng K Calianese D Mehta D Gadiyar V. Axl and mertk receptors cooperate to promote breast cancer progression by combined oncogenic signaling and evasion of host antitumor immunity. Cancer Res (2021) 81(3):698–712. doi: 10.1158/0008-5472.CAN-20-2066 33239426 PMC9999365

[43] Kasikara C Davra V Calianese D Geng K Spires TE Quigley M. Pan-TAM tyrosine kinase inhibitor BMS-777607 enhances anti-PD-1 mAb efficacy in a murine model of triple-negative breast cancer. Cancer Res (2019) 79(10):2669–83. doi: 10.1158/0008-5472.CAN-18-2614 30877108

[44] Cohen PL Caricchio R Abraham V Camenisch TD Jennette JC Roubey RA. Delayed apoptotic cell clearance and lupus-like autoimmunity in mice lacking the c-mer membrane tyrosine kinase. J Exp Med (2002) 196(1):135–40. doi: 10.1084/jem.20012094 PMC2194017 12093878

[45] Nishi C Toda S Segawa K Nagata S. Tim4- and MerTK-mediated engulfment of apoptotic cells by mouse resident peritoneal macrophages. Mol Cell Biol (2014) 34(8):1512–20. doi: 10.1128/MCB.01394-13 PMC3993587 24515440

[46] Nishi C Yanagihashi Y Segawa K Nagata S. MERTK tyrosine kinase receptor together with TIM4 phosphatidylserine receptor mediates distinct signal transduction pathways for efferocytosis and cell proliferation. J Biol Chem (2019) 294(18):7221–30. doi: 10.1074/jbc.RA118.006628 PMC6509494 30846565

[47] Gadd MS Testa A Lucas X Chan KH Chen W Lamont DJ. Structural basis of PROTAC cooperative recognition for selective protein degradation. Nat Chem Biol (2017) 13(5):514–21. doi: 10.1038/nchembio.2329 PMC5392356 28288108

[48] Akalu YT Mercau ME Ansems M Hughes LD Nevin J Alberto EJ. Tissue-specific modifier alleles determine mertk loss-of-function traits. Elife (2022) 11. doi: 10.7554/eLife.80530 PMC9433089 35969037

[49] Camenisch TD Koller BH Earp HS Matsushima GK. A novel receptor tyrosine kinase, mer, inhibits TNF-alpha production and lipopolysaccharide-induced endotoxic shock. J Immunol (1999) 162(6):3498–503. doi: 10.4049/jimmunol.162.6.3498 10092806

[50] Png KJ Halberg N Yoshida M Tavazoie SF. A microRNA regulon that mediates endothelial recruitment and metastasis by cancer cells. Nature (2011) 481(7380):190–4. doi: 10.1038/nature10661 22170610

[51] Fadok VA Bratton DL Konowal A Freed PW Westcott JY Henson PM. Macrophages that have ingested apoptotic cells *in vitro* inhibit proinflammatory cytokine production through autocrine/paracrine mechanisms involving TGF-beta, PGE2, and PAF. J Clin Invest (1998) 101(4):890–8. doi: 10.1172/JCI1112 PMC508637 9466984

[52] Cai B Kasikara C Doran AC Ramakrishnan R Birge RB Tabas I. MerTK signaling in macrophages promotes the synthesis of inflammation resolution mediators by suppressing CaMKII activity. Sci Signal (2018) 11(549). doi: 10.1126/scisignal.aar3721 PMC6171110 30254055

[53] Cai B Thorp EB Doran AC Subramanian M Sansbury BE Lin CS. MerTK cleavage limits proresolving mediator biosynthesis and exacerbates tissue inflammation. Proc Natl Acad Sci U S A (2016) 113(23):6526–31. doi: 10.1073/pnas.1524292113 PMC4988577 27199481

[54] Zizzo G Cohen PL. The PPAR-gamma antagonist GW9662 elicits differentiation of M2c-like cells and upregulation of the MerTK/Gas6 axis: a key role for PPAR-gamma in human macrophage polarization. J Inflammation (Lond) (2015) 12:36. doi: 10.1186/s12950-015-0081-4 PMC4429687 25972766

[55] Zizzo G Hilliard BA Monestier M Cohen PL. Efficient clearance of early apoptotic cells by human macrophages requires M2c polarization and MerTK induction. J Immunol (2012) 189(7):3508–20. doi: 10.4049/jimmunol.1200662 PMC3465703 22942426

[56] Zizzo G Guerrieri J Dittman LM Merrill JT Cohen PL. Circulating levels of soluble MER in lupus reflect M2c activation of monocytes/macrophages, autoantibody specificities and disease activity. Arthritis Res Ther (2013) 15(6):R212. doi: 10.1186/ar4407 24325951 PMC3978923

[57] Zhou Y Fei M Zhang G Liang WC Lin W Wu Y. Blockade of the phagocytic receptor MerTK on tumor-associated macrophages enhances P2X7R-dependent STING activation by tumor-derived cGAMP. Immunity (2020) 52(2):357–73 e9. doi: 10.1016/j.immuni.2020.01.014 32049051

[58] Lindsay RS Whitesell JC Dew KE Rodriguez E Sandor AM Tracy D. MERTK on mononuclear phagocytes regulates T cell antigen recognition at autoimmune and tumor sites. J Exp Med (2021) 218(10). doi: 10.1084/jem.20200464 PMC8383814 34415994

[59] Morse C Tabib T Sembrat J Buschur KL Bittar HT Valenzi E. Proliferating SPP1/MERTK-expressing macrophages in idiopathic pulmonary fibrosis. Eur Respir J (2019) 54(2). doi: 10.1183/13993003.02441-2018 PMC8025672 31221805

