# Supplementary material for: Targeted degradation of MERTK and other TAM receptor paralogs by heterobifunctional targeted protein degraders
Source: Front Immunol. 2023 Jul 20;14:1135373. doi: 10.3389/fimmu.2023.1135373 (PMC10397400; doi:10.3389/fimmu.2023.1135373)
Supplement: Supplementary Figure 1 — (A) Proteomic degradation profiles of KT-652 and KT-978 showed that multiple kinases including Aurora Kinase A, CDK6 (Cyclin Dependent Kinase1), AAK1 (Adaptor Associated protein Kinase 1), GAK and SIK2 (Salt Inducible Kinase) were targeted by KT-652, whereas these off- target effects were absent for KT-978. This proteomics analysis assesses selectivity of our degrader compounds against ~8,000 human proteins. However, MERTK, AXL and TYRO3 remained undetected due to their low abundance (B) A histogram of Jurkat transcriptome based on CCLE data showing transcript levels (noted in parentheses) for TAM family members and CRBN. (C) A scatterplot comparing RNA and protein levels for TAMs & CRBN across CCLE lines. Correlation values are noted in blue. (D) A tabulated gene expression summary of TAMs & CRBN across CCLE lines based on DepMap RNA and protein data. [file Image_1.pdf]

Supplemental figure 1.

A

Proteomic degradation profiles for KT-652 and KT-978.

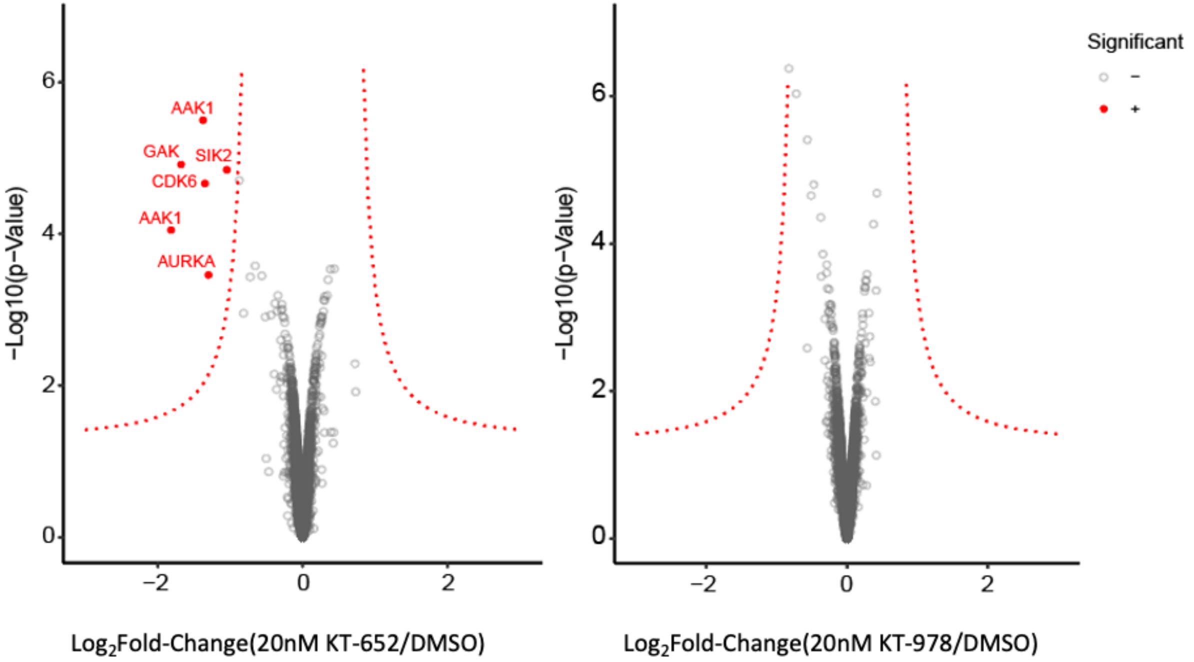

B

Histogram of Jurkat transcriptome (source: CCLE) Transcript levels noted for selected genes

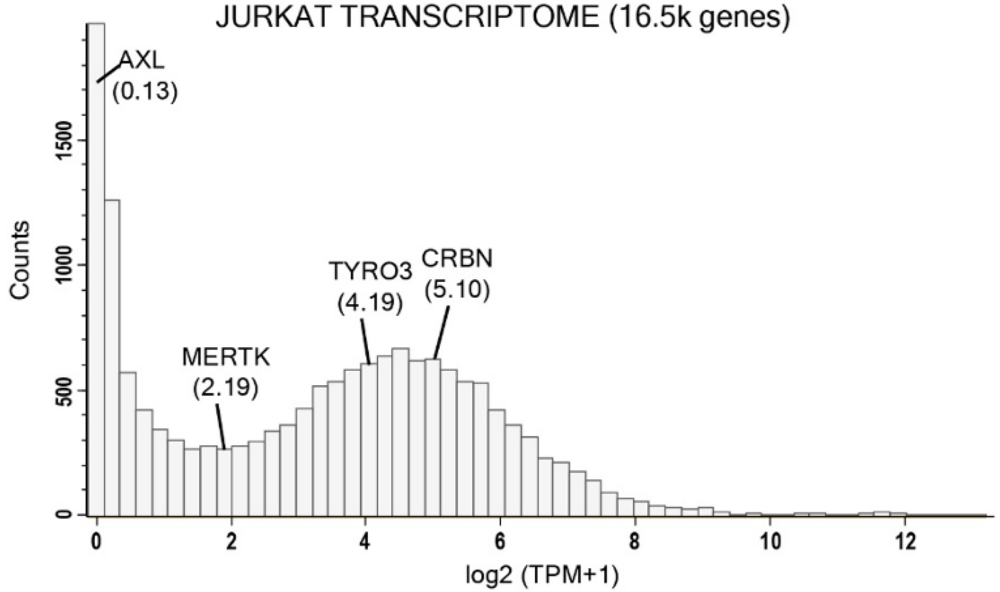

C

RNA vs Protein levels for TAMs & CRBN across CCLE lines

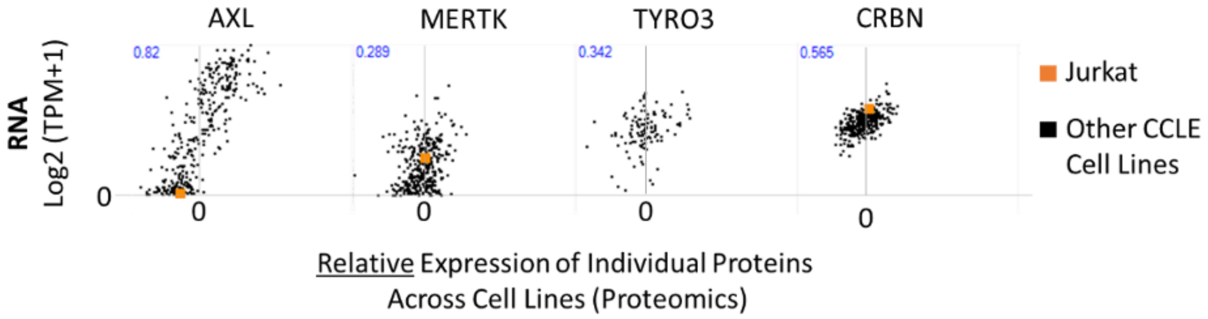

D

Gene expression of TAMs and CRBN in DepMap

Gene	Transcript (log 2 TPM+1)	% CCLE lines with RNA & Protein pair	RNA & Protein correlation	Protein abundance in Jurkat
AXL	0.13	94	0.82	Very Low
MERTK	2.19	100	0.29	Low
TYRO3	4.19	31	0.34	Not detected
CRBN	5.1	100	0.56	Medium
