# Supplementary material for: Targeted degradation of MERTK and other TAM receptor paralogs by heterobifunctional targeted protein degraders
Source: Front Immunol. 2023 Jul 20;14:1135373. doi: 10.3389/fimmu.2023.1135373 (PMC10397400; doi:10.3389/fimmu.2023.1135373)

Supplemental figure 2. Degradation of MERTK in spleens at different time points from mice treated with KTX-652.

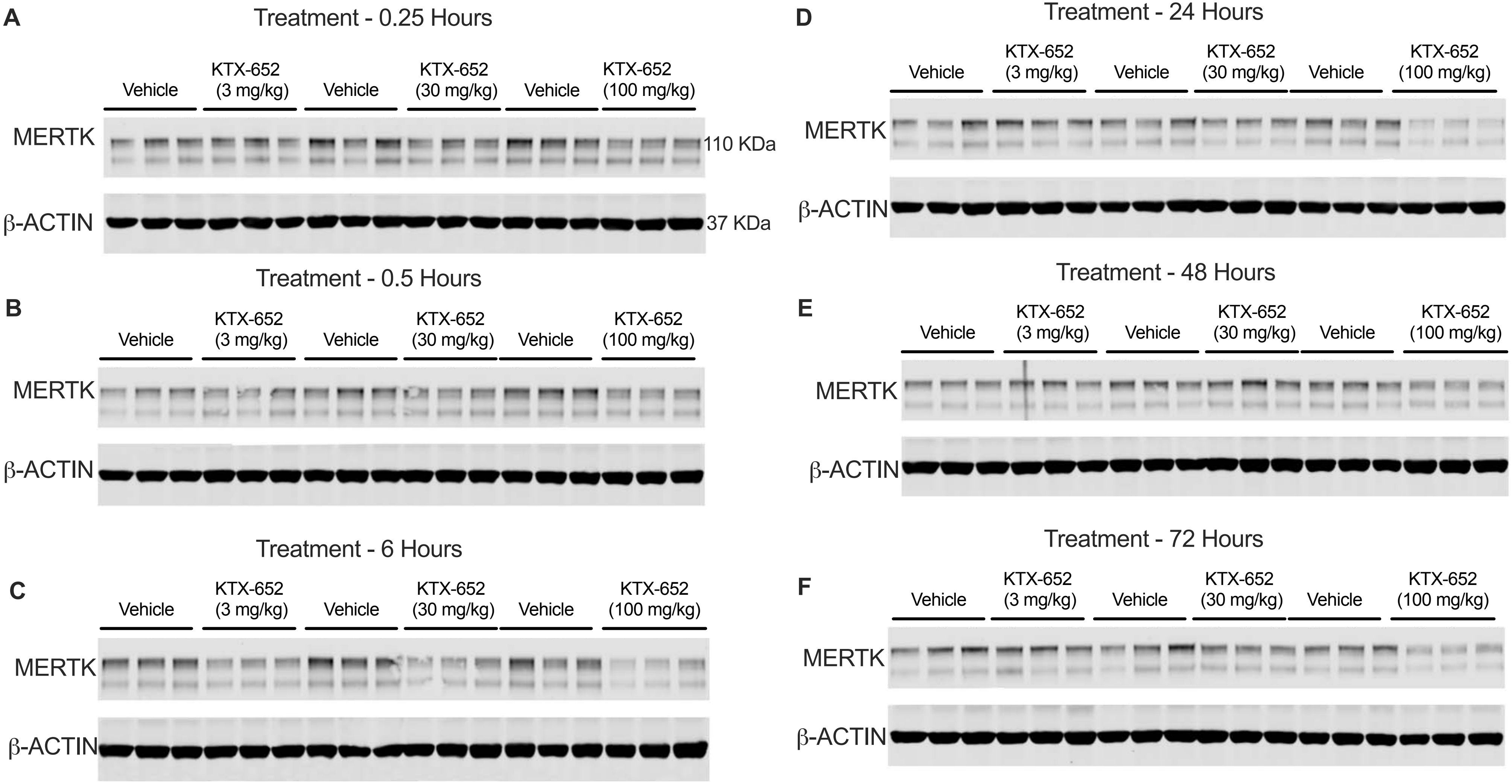

Supplement: Supplementary Figure 2 — Western blot images representation of MERTK degradation in spleens of mice treated with KTX-652 at 3 mg/kg, 30 mg/kg and 100 mg/kg doses, at different time points, 0.25 hours (A), 0.5 hours, (B), 6 hours (C), 24 hours (D), 48 hours (E), and 72 hours (F). [file Image_2.pdf]
