# Supplementary figures and images for: Targeted degradation of MERTK and other TAM receptor paralogs by heterobifunctional targeted protein degraders
Source: Front Immunol. 2023 Jul 20;14:1135373. doi: 10.3389/fimmu.2023.1135373 (PMC10397400; doi:10.3389/fimmu.2023.1135373)

Supplemental figure 3. Degradation of EGFR/MERTK receptor by KTX-978 and KTX-214

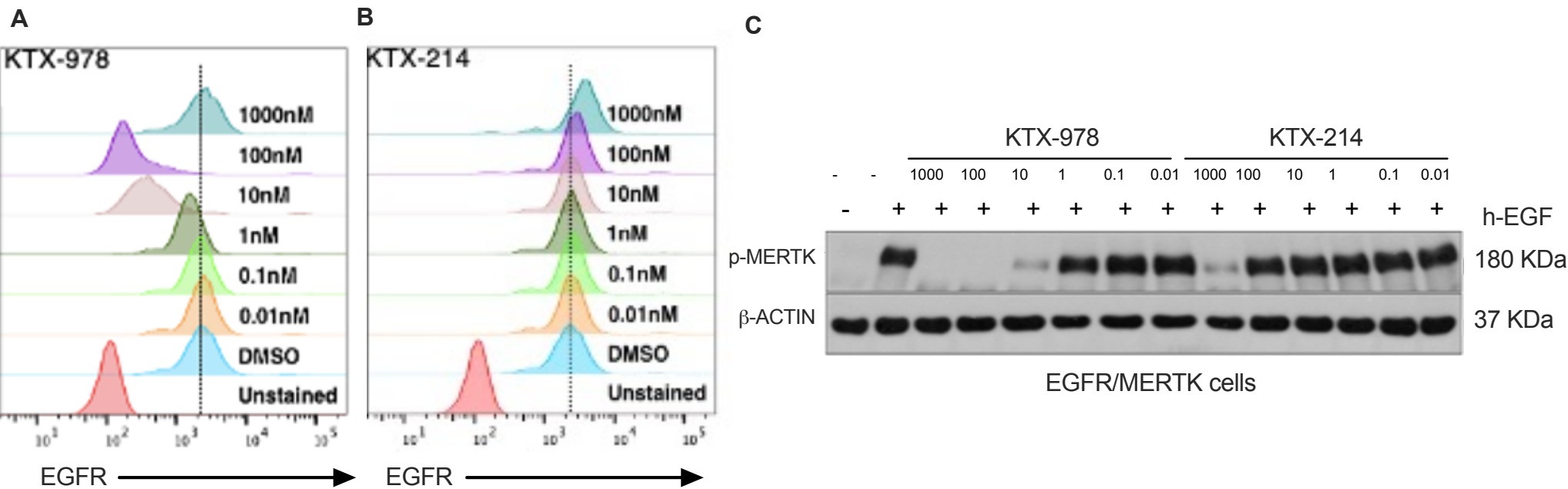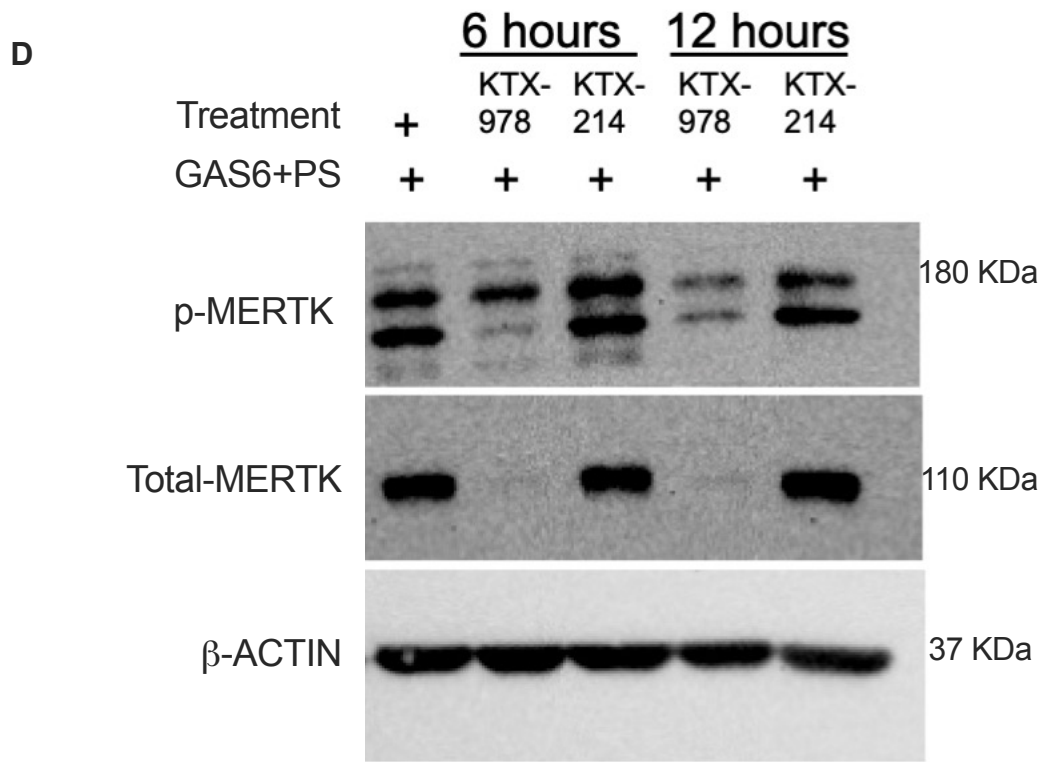

Supplement: Supplementary Figure 3 — Flow cytometry histograms show that KTX-978 (A) degrades MERTK on EGFR-MERTK chimeric cell lines at nanomolar concentrations, whereas KTX-214 (B) demonstrates no degradation. Effect of KTX-978 is further shown by reduced downstream activation of p-MERTK when EGFR-MERTK chimeric cell lines are activated by recombinant h-EGF (C). (D) MERTK activation on BMDMs is shown by p-MERTK. Total MERTK does not rebound after 6 or 12 hours of removal of KTX-978 and is reflected in reduced p-MERTK activation. [file Image_3.pdf]
